# 5-(4-Fluoro­phen­yl)-4-(4-pyrid­yl)-1,3-oxazol-2-amine

**DOI:** 10.1107/S1600536810009189

**Published:** 2010-03-24

**Authors:** Pierre Koch, Dieter Schollmeyer, Stefan Laufer

**Affiliations:** a Institute of Pharmacy, Department of Pharmaceutical and Medicinal Chemistry, Eberhard-Karls-University Tübingen, Auf der Morgenstelle 8, 72076 Tübingen, Germany; b Department of Organic Chemistry, Johannes Gutenberg-University Mainz, Duesbergweg 10-14, 55099 Mainz, Germany

## Abstract

In the crystal structure of the title compound, C_14_H_10_FN_3_O, the plane of the isoxazole ring makes dihedral angles of 35.72 (9) and 30.00 (9)°, respectively, with those of the 4-fluoro­phenyl and pyridine rings. The plane of the 4-fluoro­phenyl ring makes a dihedral angle of 45.85 (8)° with that of the pyridine ring. The crystal structure is stabilized by inter­molecular N—H⋯N hydrogen bonding. The two types of hydrogen bonds result in two chains, extending along the *a* axis, which are related by centres of symmetry.

## Related literature

For the biological activity of pyridinyloxazoles, see: Peifer *et al.* (2006 ▶). For p38α MAP kinase inhibitors having a vicinal 4-fluoro­phen­yl/pyridin-4-yl system connected to a five-membered heterocyclic core, see: Abu Thaher *et al.* (2009 ▶). 
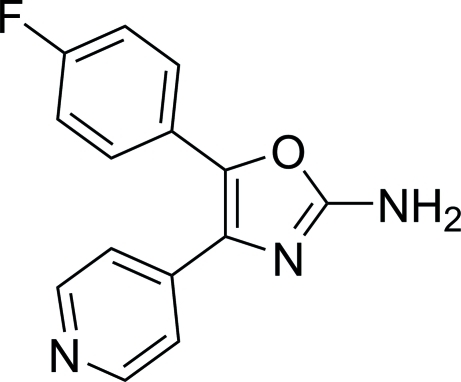


## Experimental

### 

#### Crystal data

C_14_H_10_FN_3_O
*M* *_r_* = 255.25
Orthorhombic, 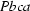

*a* = 10.1017 (4) Å
*b* = 8.3889 (8) Å
*c* = 29.127 (2) Å
*V* = 2468.3 (3) Å^3^
*Z* = 8
Cu *K*α radiation
μ = 0.84 mm^−1^
*T* = 193 K
0.40 × 0.30 × 0.10 mm

#### Data collection

Enraf–Nonius CAD-4 diffractometer
Absorption correction: ψ scan (*CORINC*; Dräger & Gattow, 1971 ▶) *T*_min_ = 0.899, *T*_max_ = 0.997
2331 measured reflections
2331 independent reflections
1859 reflections with *I* > 2σ(*I*)
3 standard reflections every 60 min  intensity decay: 2%

#### Refinement

*R*[*F* ^2^ > 2σ(*F* ^2^)] = 0.043
*wR*(*F* ^2^) = 0.119
*S* = 1.05
2331 reflections
173 parameters
H-atom parameters constrained
Δρ_max_ = 0.20 e Å^−3^
Δρ_min_ = −0.17 e Å^−3^

### 

Data collection: *CAD-4 Software* (Enraf–Nonius, 1989 ▶); cell refinement: *CAD-4 Software*; data reduction: *CORINC* (Dräger & Gattow, 1971 ▶); program(s) used to solve structure: *SIR97* (Altomare *et al.*, 1999 ▶); program(s) used to refine structure: *SHELXL97* (Sheldrick, 2008 ▶); molecular graphics: *PLATON* (Spek, 2009 ▶); software used to prepare material for publication: *PLATON*.

## Supplementary Material

Crystal structure: contains datablocks I, global. DOI: 10.1107/S1600536810009189/nc2179sup1.cif

Structure factors: contains datablocks I. DOI: 10.1107/S1600536810009189/nc2179Isup2.hkl

Additional supplementary materials:  crystallographic information; 3D view; checkCIF report

## Figures and Tables

**Table 1:** Hydrogen-bond geometry (Å, °)

*D*—H⋯*A*	*D*—H	H⋯*A*	*D*⋯*A*	*D*—H⋯*A*
N1—H1*A*⋯N4^i^	1.00	1.99	2.983 (2)	175
N1—H1*B*⋯N15^ii^	0.91	2.03	2.929 (2)	165

Symmetry codes: (i) 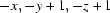; (ii) 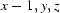.

**Fig. 1.**
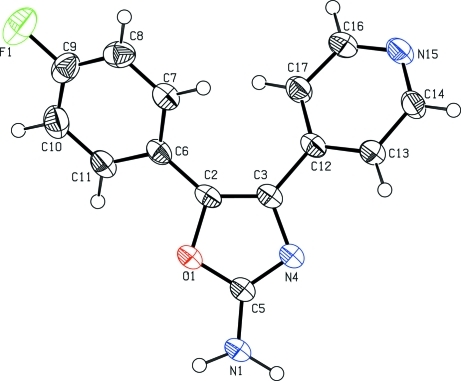
Molecular structure of the title compound with labelling and displacement ellipsoids are drawn at the 50% propability level.

**Fig. 2.**
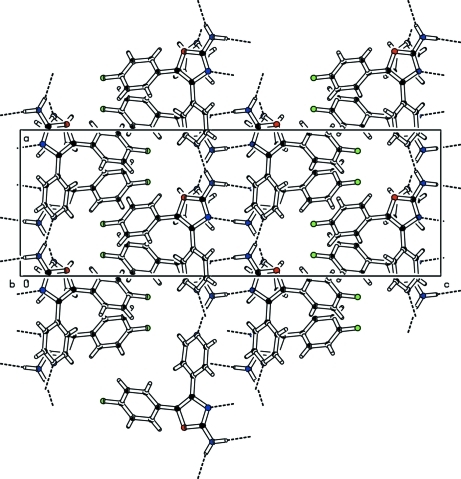
Crystal structure of the title compound with view along the b-axis (hydrogen bonding is shown with dashed lines).

## supplementary crystallographic information

### Comment

Compounds having a vicinal 4-fluorophenyl/pyridin-4-yl system connected to a five-membered heterocyclic core have been considered to be potential p38α MAP kinase inhibitors (Abu Thaher *et al.* 2009, Peifer *et al.* 2006).

The microwave-assisted reaction of 2-bromo-2-(4-fluorophenyl)-1-(pyridin-4-yl)ethanone hydrobromide and urea could gave two products, namely 5-(4-flouorophenyl)-4-(pyridin-4-yl)oxazol-2-amine and 4-(4-fluorophenyl)-5-(pyridin-4-yl)-1,3-dihydroimidazol-2-one. The structure determination was undertaken to identify the obtained product and showed that only 5-(4-flouorophenyl)-4-(pyridin-4-yl)oxazol-2-amine was formed in the reaction above-mentioned.

In the crystal structure of the title compound, the isoxazole ring makes dihedral angles of 35.72 (9)° and 30.00 (9)° to the 4-fluorophenyl ring and the pyridine ring, respectively (Figure 1). The 4-fluorophenyl ring makes dihedral angles of 45.85 (8)° to the pyridine ring.

The crystal packing (Figure 2) shows that the amino function acts an a hydrogen bond donor forming hydrogen bonds to the nitrogen atom of the pyridine ring and to the nitrogen atom of the oxale ring of two different molecules. The lenght of the hydrogen bonds is 1.99 Å and 2.03 Å, respectively (Table 1). The two types of hydrogen bonds result in two chains that elongate along the a-axis which are related by centres of symmetry.

### Experimental

2-Bromo-2-(4-fluorophenyl)-1-(pyridin-4-yl)ethanone hydrobromide (150 mg, 0.40 mmol), urea (24 mg, 0.40 mmol) and DMF (1 ml) were combined in a reaction vial. The reaction vessel was heated in a CEM microwave reactor for 10 min at 433 K (initial power 250 W) and afterwards the vessel was cooled down to room temperature by a stream of compressed air. Water and ethyl acetate were added and the organic layer was separated. This layer was washed with water (3x), dried over Na_2_SO_4_ and concentrated in vacuo. The yellow residue was suspended twice with DCM/EtOH 95-5, filtered and finally dried. Yield 83 mg (81 %). Suitable crystals of the title compound for X-ray were obtained by slow evaporation at 298 K of a solution of methanol.

### Refinement

Hydrogen atoms attached to carbons were placed at calculated positions with C—H = 0.95 Å (aromatic) or 0.98–0.99 Å (*sp*^3^ C-atom). The H atoms attached to N1 were located in diff. Fourier maps. All H atoms were refined in the riding-model approximation with isotropic displacement parameters (set at 1.2–1.5 times of the *U*_eq_ of the parent atom).

### Crystal data

**Table:**

C_14_H_10_FN_3_O	*F*(000) = 1056
*M**_r_* = 255.25	*D*_x_ = 1.374 Mg m^−^^3^
Orthorhombic, *P**b**c**a*	Cu *K*α radiation, λ = 1.54178 Å
Hall symbol: -P 2ac 2ab	Cell parameters from 25 reflections
*a* = 10.1017 (4) Å	θ = 30–46°
*b* = 8.3889 (8) Å	µ = 0.84 mm^−^^1^
*c* = 29.127 (2) Å	*T* = 193 K
*V* = 2468.3 (3) Å^3^	Plate, yellow
*Z* = 8	0.40 × 0.30 × 0.10 mm

### Data collection

**Table:**

Enraf–Nonius CAD-4 diffractometer	1859 reflections with *I* > 2σ(*I*)
Radiation source: rotating anode	*R*_int_ = 0.0000
graphite	θ_max_ = 69.9°, θ_min_ = 3.0°
ω/2θ scans	*h* = 0→12
Absorption correction: ψ scan (CORINC; Dräger & Gattow, 1971)	*k* = 0→10
*T*_min_ = 0.899, *T*_max_ = 0.997	*l* = −35→0
2331 measured reflections	3 standard reflections every 60 min
2331 independent reflections	intensity decay: 2%

### Refinement

**Table:**

Refinement on *F*^2^	Secondary atom site location: difference Fourier map
Least-squares matrix: full	Hydrogen site location: inferred from neighbouring sites
*R*[*F*^2^ > 2σ(*F*^2^)] = 0.043	H-atom parameters constrained
*wR*(*F*^2^) = 0.119	*w* = 1/[σ^2^(*F*_o_^2^) + (0.0607*P*)^2^ + 0.5691*P*] where *P* = (*F*_o_^2^ + 2*F*_c_^2^)/3
*S* = 1.05	(Δ/σ)_max_ = 0.001
2331 reflections	Δρ_max_ = 0.20 e Å^−^^3^
173 parameters	Δρ_min_ = −0.17 e Å^−^^3^
0 restraints	Extinction correction: *SHELXL97* (Sheldrick, 2008), Fc^*^=kFc[1+0.001xFc^2^λ^3^/sin(2θ)]^-1/4^
Primary atom site location: structure-invariant direct methods	Extinction coefficient: 0.00056 (12)

### Special details

**Table:**

Geometry. All esds (except the esd in the dihedral angle between two l.s. planes) are estimated using the full covariance matrix. The cell esds are taken into account individually in the estimation of esds in distances, angles and torsion angles; correlations between esds in cell parameters are only used when they are defined by crystal symmetry. An approximate (isotropic) treatment of cell esds is used for estimating esds involving l.s. planes.
Refinement. Refinement of *F*^2^ against ALL reflections. The weighted *R*-factor wR and goodness of fit *S* are based on *F*^2^, conventional *R*-factors *R* are based on *F*, with *F* set to zero for negative *F*^2^. The threshold expression of *F*^2^ > σ(*F*^2^) is used only for calculating *R*-factors(gt) etc. and is not relevant to the choice of reflections for refinement. *R*-factors based on *F*^2^ are statistically about twice as large as those based on *F*, and *R*- factors based on ALL data will be even larger.

### Fractional atomic coordinates and isotropic or equivalent isotropic displacement parameters (Å^2^)

**Table:**

	*x*	*y*	*z*	*U*_iso_*/*U*_eq_	
F1	0.14358 (14)	0.10350 (16)	0.19761 (4)	0.0644 (4)	
N1	−0.13607 (15)	0.4430 (3)	0.45720 (6)	0.0541 (5)	
H1A	−0.1264	0.4860	0.4890	0.081*	
H1B	−0.2133	0.4266	0.4415	0.081*	
O1	−0.03766 (11)	0.36315 (17)	0.39018 (4)	0.0382 (3)	
C2	0.09228 (16)	0.3491 (2)	0.37416 (6)	0.0329 (4)	
C3	0.17254 (16)	0.3972 (2)	0.40860 (6)	0.0321 (4)	
N4	0.09727 (14)	0.44405 (19)	0.44663 (5)	0.0362 (4)	
C5	−0.02508 (16)	0.4204 (2)	0.43365 (6)	0.0378 (4)	
C6	0.10541 (15)	0.2869 (2)	0.32790 (6)	0.0325 (4)	
C7	0.20470 (17)	0.3409 (2)	0.29845 (6)	0.0371 (4)	
H7	0.2644	0.4208	0.3087	0.044*	
C8	0.21765 (18)	0.2803 (2)	0.25471 (6)	0.0423 (5)	
H8	0.2856	0.3175	0.2348	0.051*	
C9	0.1303 (2)	0.1652 (2)	0.24045 (6)	0.0435 (5)	
C10	0.0300 (2)	0.1098 (2)	0.26787 (7)	0.0463 (5)	
H10	−0.0300	0.0314	0.2569	0.056*	
C11	0.01776 (17)	0.1703 (2)	0.31191 (6)	0.0394 (4)	
H11	−0.0507	0.1324	0.3314	0.047*	
C12	0.31809 (16)	0.4044 (2)	0.41163 (6)	0.0316 (4)	
C13	0.37771 (16)	0.5163 (2)	0.43992 (6)	0.0348 (4)	
H13	0.3254	0.5891	0.4571	0.042*	
C14	0.51470 (17)	0.5205 (2)	0.44293 (6)	0.0395 (4)	
H14	0.5539	0.5991	0.4621	0.047*	
N15	0.59471 (15)	0.4203 (2)	0.42047 (5)	0.0439 (4)	
C16	0.53651 (17)	0.3118 (3)	0.39376 (7)	0.0434 (5)	
H16	0.5915	0.2388	0.3777	0.052*	
C17	0.40105 (17)	0.2992 (2)	0.38802 (6)	0.0371 (4)	
H17	0.3649	0.2201	0.3683	0.045*	

### Atomic displacement parameters (Å^2^)

**Table:**

	*U*^11^	*U*^22^	*U*^33^	*U*^12^	*U*^13^	*U*^23^
F1	0.0878 (10)	0.0654 (8)	0.0399 (6)	0.0139 (7)	−0.0042 (6)	−0.0098 (6)
N1	0.0211 (7)	0.0942 (14)	0.0470 (9)	0.0003 (8)	0.0007 (7)	−0.0199 (9)
O1	0.0204 (6)	0.0547 (8)	0.0394 (7)	0.0007 (5)	−0.0015 (5)	−0.0051 (6)
C2	0.0199 (8)	0.0389 (9)	0.0400 (9)	0.0008 (7)	0.0013 (7)	0.0024 (7)
C3	0.0233 (8)	0.0345 (8)	0.0383 (9)	−0.0002 (7)	0.0002 (7)	0.0010 (7)
N4	0.0236 (7)	0.0476 (9)	0.0375 (8)	−0.0002 (6)	−0.0009 (6)	−0.0051 (7)
C5	0.0237 (8)	0.0513 (11)	0.0384 (10)	0.0011 (8)	−0.0010 (7)	−0.0059 (8)
C6	0.0229 (7)	0.0369 (9)	0.0378 (9)	0.0032 (7)	−0.0047 (7)	0.0036 (7)
C7	0.0294 (8)	0.0396 (9)	0.0422 (10)	0.0006 (7)	−0.0020 (7)	0.0017 (8)
C8	0.0394 (10)	0.0465 (11)	0.0410 (10)	0.0096 (8)	0.0044 (8)	0.0064 (9)
C9	0.0513 (11)	0.0455 (11)	0.0339 (9)	0.0147 (9)	−0.0062 (8)	−0.0038 (8)
C10	0.0401 (10)	0.0481 (12)	0.0507 (11)	0.0015 (9)	−0.0138 (9)	−0.0063 (9)
C11	0.0258 (8)	0.0480 (11)	0.0444 (10)	−0.0017 (8)	−0.0047 (7)	−0.0005 (9)
C12	0.0222 (8)	0.0362 (9)	0.0364 (9)	0.0005 (7)	−0.0014 (7)	0.0050 (7)
C13	0.0269 (8)	0.0406 (10)	0.0368 (9)	−0.0008 (7)	0.0002 (7)	−0.0003 (8)
C14	0.0279 (8)	0.0510 (11)	0.0396 (10)	−0.0071 (8)	−0.0049 (7)	0.0010 (9)
N15	0.0245 (7)	0.0632 (11)	0.0441 (9)	−0.0009 (7)	−0.0029 (6)	0.0010 (8)
C16	0.0274 (9)	0.0548 (12)	0.0480 (11)	0.0074 (8)	−0.0006 (8)	−0.0032 (9)
C17	0.0281 (9)	0.0391 (10)	0.0442 (10)	0.0020 (7)	−0.0034 (7)	−0.0023 (8)

### Geometric parameters (Å, °)

**Table:**

F1—C9	1.358 (2)	C8—H8	0.9500
N1—C5	1.328 (2)	C9—C10	1.372 (3)
N1—H1A	0.9994	C10—C11	1.385 (3)
N1—H1B	0.9144	C10—H10	0.9500
O1—C5	1.360 (2)	C11—H11	0.9500
O1—C2	1.3981 (19)	C12—C13	1.386 (2)
C2—C3	1.351 (2)	C12—C17	1.398 (2)
C2—C6	1.451 (2)	C13—C14	1.387 (2)
C3—N4	1.400 (2)	C13—H13	0.9500
C3—C12	1.474 (2)	C14—N15	1.337 (3)
N4—C5	1.308 (2)	C14—H14	0.9500
C6—C7	1.395 (2)	N15—C16	1.334 (3)
C6—C11	1.399 (2)	C16—C17	1.383 (2)
C7—C8	1.378 (3)	C16—H16	0.9500
C7—H7	0.9500	C17—H17	0.9500
C8—C9	1.372 (3)		
			
C5—N1—H1A	116.6	F1—C9—C8	118.89 (18)
C5—N1—H1B	116.2	C10—C9—C8	122.51 (17)
H1A—N1—H1B	126.9	C9—C10—C11	118.74 (18)
C5—O1—C2	104.64 (13)	C9—C10—H10	120.6
C3—C2—O1	106.88 (15)	C11—C10—H10	120.6
C3—C2—C6	137.89 (15)	C10—C11—C6	120.54 (18)
O1—C2—C6	115.22 (14)	C10—C11—H11	119.7
C2—C3—N4	110.20 (14)	C6—C11—H11	119.7
C2—C3—C12	130.92 (17)	C13—C12—C17	117.35 (15)
N4—C3—C12	118.87 (15)	C13—C12—C3	119.78 (16)
C5—N4—C3	104.01 (14)	C17—C12—C3	122.85 (16)
N4—C5—N1	128.84 (17)	C12—C13—C14	119.23 (17)
N4—C5—O1	114.27 (15)	C12—C13—H13	120.4
N1—C5—O1	116.89 (15)	C14—C13—H13	120.4
C7—C6—C11	118.51 (17)	N15—C14—C13	123.78 (18)
C7—C6—C2	121.31 (15)	N15—C14—H14	118.1
C11—C6—C2	120.18 (16)	C13—C14—H14	118.1
C8—C7—C6	121.12 (17)	C16—N15—C14	116.61 (16)
C8—C7—H7	119.4	N15—C16—C17	123.99 (18)
C6—C7—H7	119.4	N15—C16—H16	118.0
C9—C8—C7	118.57 (18)	C17—C16—H16	118.0
C9—C8—H8	120.7	C16—C17—C12	119.03 (17)
C7—C8—H8	120.7	C16—C17—H17	120.5
F1—C9—C10	118.59 (19)	C12—C17—H17	120.5
			
C5—O1—C2—C3	0.36 (19)	C7—C8—C9—F1	−179.13 (16)
C5—O1—C2—C6	179.09 (15)	C7—C8—C9—C10	0.8 (3)
O1—C2—C3—N4	−0.7 (2)	F1—C9—C10—C11	178.78 (17)
C6—C2—C3—N4	−179.0 (2)	C8—C9—C10—C11	−1.1 (3)
O1—C2—C3—C12	178.41 (17)	C9—C10—C11—C6	0.6 (3)
C6—C2—C3—C12	0.1 (4)	C7—C6—C11—C10	0.3 (3)
C2—C3—N4—C5	0.7 (2)	C2—C6—C11—C10	−179.70 (16)
C12—C3—N4—C5	−178.50 (16)	C2—C3—C12—C13	151.88 (19)
C3—N4—C5—N1	178.7 (2)	N4—C3—C12—C13	−29.1 (2)
C3—N4—C5—O1	−0.5 (2)	C2—C3—C12—C17	−30.1 (3)
C2—O1—C5—N4	0.1 (2)	N4—C3—C12—C17	148.94 (17)
C2—O1—C5—N1	−179.15 (18)	C17—C12—C13—C14	1.0 (3)
C3—C2—C6—C7	−37.0 (3)	C3—C12—C13—C14	179.18 (17)
O1—C2—C6—C7	144.77 (16)	C12—C13—C14—N15	−1.1 (3)
C3—C2—C6—C11	142.9 (2)	C13—C14—N15—C16	0.3 (3)
O1—C2—C6—C11	−35.3 (2)	C14—N15—C16—C17	0.5 (3)
C11—C6—C7—C8	−0.6 (3)	N15—C16—C17—C12	−0.6 (3)
C2—C6—C7—C8	179.34 (16)	C13—C12—C17—C16	−0.3 (3)
C6—C7—C8—C9	0.1 (3)	C3—C12—C17—C16	−178.35 (18)

### Hydrogen-bond geometry (Å, °)

**Table:**

*D*—H···*A*	*D*—H	H···*A*	*D*···*A*	*D*—H···*A*
N1—H1A···N4^i^	1.00	1.99	2.983 (2)	175
N1—H1B···N15^ii^	0.91	2.03	2.929 (2)	165

Symmetry codes: (i) −*x*, −*y*+1, −*z*+1; (ii) *x*−1, *y*, *z*.
